# Biodegradable Stents in Companion Animals: A Systematic Scoping Review

**DOI:** 10.1155/vmi/6405530

**Published:** 2025-02-26

**Authors:** Daiana R. Cardoso, Alexandre Barros, André Meneses, João F. Requicha

**Affiliations:** ^1^I-MVET Research in Veterinary Medicine, Faculty of Veterinary Medicine, Lusófona University—Lisbon University Centre, Lisbon, Portugal; ^2^Department of Veterinary Sciences, University of Trás-os-Montes and Alto Douro, Vila Real, Portugal; ^3^Associate Laboratory for Animal and Veterinary Sciences (AL4AnimalS), Lisbon, Portugal; ^4^Veterinary and Animal Research Center (CECAV), Lusófona University Center, Lisbon, Portugal; ^5^Hydrumedical S.A., Guimarães, Portugal; ^6^Animal and Veterinary Research Centre (CECAV), University of Trás-os-Montes e Alto Douro (UTAD), Vila Real, Portugal

**Keywords:** absorbable implants, animal models, biomaterials, interventional urology, medical devices, veterinary medicine

## Abstract

**Objective:** Describe the existing scientific literature regarding the feasibility and behavior of biodegradable stents (BDSs) in companion animals.

**Study Design:** Systematic scoping review.

**Methods:** A literature search was conducted in accordance with the Preferred Reporting Items for Systematic Reviews and Meta-Analyses Extension for Scoping Reviews (PRISMA-ScR) and followed the recommendations of the Cochrane Collaboration. The search was performed by using PubMed, Web of Science, and Scopus databases, focusing on BDS usage in companion animals' urinary, respiratory, and digestive systems including choledochal duct.

**Results:** In total, 233 articles were identified but only 21 fulfilled the inclusion criteria. Ninety percent (*n* = 19) of the investigation was conducted in animal models aiming translational to humans, and only two studies involved clinical cases. Regarding application, 42.9% was focused on the urinary system, 19% assessed vascular stenting, 14.3% esophageal stenting, 19% choledochal duct stenting, and 4.8% tracheal stenting. Polylactic acid (PLA) was the most used biomaterial.

**Conclusion:** This review summarized the use of BDS in companion animals, highlighting that most studies were conducted in animals without clinical disease, with most reporting a low incidence of self-limiting complications.

**Clinical Significance:** This review underscores the potential impact of BDS on companion animals. Further research is necessary to explore BDS's full potential in small animal medicine.

## 1. Introduction

The first use of stents in human medicine was reported in 1986, and their application has since become widespread [1]. A stent is a cylindrical medical device designed to dilate intraluminal strictures, restore or maintain lumen patency, and preserve physiological function [2].

The rapid development of biodegradable materials for biomedical applications has significantly advanced research into biodegradable stents (BDSs). For a BDS to perform effectively, several critical requirements must be met, including controlled degradation or absorption within the body, smooth delivery during compression, and sufficient radial force to dilate strictures effectively once deployed [2, 3]. Studies in humans have shown that BDS can reduce the need for repeated surgeries or endoscopic procedures for stent removal [4] while also minimizing complications such as tissue hyperplasia and stent migration compared to nonbiodegradable stents (non-BDSs) [5].

In companion animals, the use of non-BDS has grown steadily since the early 21st century. These stents have been employed to treat a wide range of conditions, including nasopharyngeal stenosis, tracheal and bronchial collapse, esophageal and colonic strictures, ureteral and urethral obstructions, liver disorders such as Budd–Chiari syndrome, and cardiac diseases like pulmonary stenosis [6].

However, the clinical use of BDS in companion animals remains limited [7, 8]. Existing reports are largely derived from research in human medicine, where animal models are frequently used for translational studies.

BDSs are constructed from a wide range of materials, with polymers and metal composites standing out as the primary categories. Polymers, which are chains of repeating monomers, can be either natural or synthetic, each offering distinct advantages and limitations [9]. Synthetic polymers are particularly advantageous due to their predictable and reproducible properties, such as tensile strength, elastic modulus, and degradation rates. Compared with natural polymers like silk or cellulose, synthetic polymers also pose lower risks of toxicity, immunogenicity, and infections [9, 10].

Among synthetic polymers, polyesters have received significant attention for biomedical applications, especially in the design of BDS. Their chemical composition allows for hydrolytic degradation via de-esterification, enabling gradual breakdown and eventual resorption of the stent material within the body. A variety of polyesters, such as poly(lactic acid) (PLA), poly(glycolic acid) (PGA), poly(butylene succinate) (PBS), polycaprolactone (PCL), poly(ethylene adipate) (PEA), and poly(p-dioxanone) (PDX), have been widely used in stent development [9, 10].

To enhance material performance, different polymers are often combined to create copolymers, such as poly(lactic-coglycolic acid) (PLGA). This approach allows for the fine-tuning of stent properties, such as degradation rates and mechanical behavior. In addition, the integration of polymers with metallic frameworks has led to the development of metal-polymer composites. These composites combine the mechanical strength of metals with the controlled biodegradation properties of polymers, resulting in more durable and adaptable stents [11].

Metals such as iron (Fe), zinc (Zn), and magnesium (Mg) are commonly used in the fabrication of BDS. Among these, Mg is particularly favored due to its excellent biomechanical properties and biocompatibility. As an essential nutrient, Mg plays a vital role in many physiological processes. Moreover, Mg stents biodegrade completely without eliciting foreign body reactions, making them a highly suitable choice for biomedical applications [11].

While BDSs are gaining widespread use in human medicine, their application in veterinary medicine remains underexplored. The potential benefits and challenges of their use in companion animals are still unclear, highlighting the need for further investigation.

To address this knowledge gap, a scoping review was conducted to systematically map the existing translational and clinical research on BDSs in veterinary medicine. The review aimed to answer the research question: “What is the current state-of-the-art regarding the use of BDSs in companion animals?” Specifically, it focused on the types of biodegradable materials used, their clinical applications and outcomes, and their safety in use.

## 2. Materials and Methods

This scoping review was conducted in accordance with the Preferred Reporting Items for Systematic Reviews and Meta-Analyses Extension for Scoping Reviews (PRISMA-ScR) [12] and followed the recommendations of the Cochrane Collaboration (Cochrane Handbook for Systematic Reviews of Interventions) [13].

### 2.1. Literature Search

The literature search was conducted in February 2024 and included peer-reviewed journal articles, conference abstracts, and technical reports authored but not subject to peer review and personal communications. The search focused on studies assessing the use of BDSs in companion animals and was carried out using three major databases: Scopus, PubMed, and Web of Science.

The search was limited to studies published between January 1990 and January 2024. To capture all relevant studies, a comprehensive query was designed using MeSH entry terms combined with Boolean operators. The search strategy included the following terms: (biodegradable OR bioabsorbable) AND (stent) AND (feline OR cat OR canine OR dog). This approach was applied consistently across all databases to ensure uniformity.

### 2.2. Study Selection

Search results from the three scientific databases were imported into Mendeley Reference Manager Software for organization. After manually removing duplicates, two reviewers (DC and JR) assessed the titles and abstracts to identify studies that met the inclusion criteria. Articles were categorized as ‘suitable' or ‘not suitable.' Those clearly not meeting the eligibility criteria were excluded, while abstracts with insufficient information or deemed potentially relevant were provisionally categorized as ‘suitable' for further evaluation. Full-text articles were then obtained for all studies categorized as ‘suitable.' In cases of disagreement between reviewers, a third reviewer (AB) was consulted to resolve discrepancies. Full-text articles were reviewed by DC for inclusion in the scoping review using predefined eligibility criteria. In addition, the reference lists of these articles were examined to identify further relevant studies. The following criteria were used to determine the eligibility of studies: (i) studies involving the use of BDSs in companion animals (cats and dogs), (ii) studies identifying biomaterials applied to the urinary, respiratory, cardiovascular, or digestive systems in canine and feline models, (iii) studies published in English, Spanish, or Portuguese, and (iv) analytic observational studies (retrospective or prospective) and descriptive studies, including case series and case reports. Studies were excluded if (i) not published in English, Spanish, or Portuguese, (ii) classified as dissertations or theses, (iii) evaluated only coating effect, (iv) evaluated biodegradable material that were not stents, (v) investigated nonabsorbable stents, and (vi) the full text was not available online.

### 2.3. Data Extraction and Data Charting

A data-charting form was created to systematically extract relevant variables. The extracted data included authors, publication date, study design, case numbers, species involved, implantation site details, material specifications, degradation rates, clinical outcomes, complications, and safety considerations during application.

### 2.4. Synthesis of Results

A qualitative synthesis was conducted to analyze and organize the results, aiming to identify key patterns, trends, and gaps in the literature. Findings were grouped by the study type, implantation site, biodegradable material, degradation rate, and reported complications.

## 3. Results

**Figure 1:**
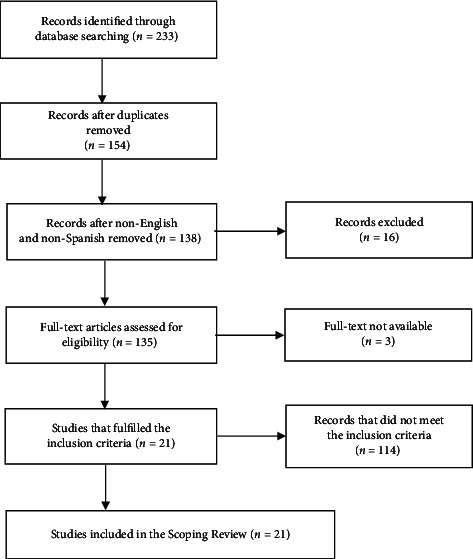
PRISMA flowchart for the study selection process in this scoping review based on searches conducted in Scopus, PubMed, and Web of Science. Exclusion criteria included studies not published in English, Spanish, or Portuguese; dissertations or theses; studies focused solely on coating effects; biodegradable materials other than stents; studies involving nonabsorbable stents; and studies where the full text was not available online.

The database search resulted in 233 references. After removing the duplicates and the non-English, non-Spanish and non-Portuguese articles, 136 were selected for the full-text review. From those, only 21 original articles fulfilled the inclusion criteria and were selected for the review. A total of 21 articles were excluded because they did not include companion animals (cats and dogs), 37 were excluded because they did not involve the use of stents, 27 were excluded because they did not use biodegradable materials, and 29 were excluded because they only studied the coating effect (Figure 1).

**Figure 2:**
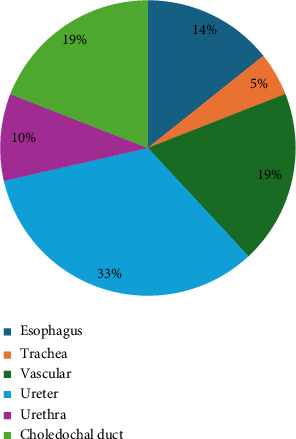
Relative distribution of biodegradable stent applications by anatomical location, expressed as a percentage of the total cases reviewed. The largest proportion of stents was applied in the choledochal duct (33%), followed by vascular structures (19%) and the ureter (19%). Applications in the esophagus accounted for 14% of the cases, while 10% of stents were used in the urethra and 5% in the trachea.

The key results of the relevant studies on BDS use in companion animals presented in Tables 1, 2, and 3. The older study is from 1997 and the more recent from 2021. Considering the type of study, 19 articles were classified as prospective, analytical, and experimental and 2 were classified as retrospective, descriptive, and observational. A total of 246 animals were included in the selected studies, including 245 dogs and one cat. Of these, nine studies (42.9%) focused on the urinary system, specifically the ureter (*n* = 7) and the urethra (*n* = 2). Four studies (19%) assessed vascular stenting, three esophageal stenting (14.3%), four choledochal duct stenting (19%), and one tracheal stenting (4.8%) (Figure 2).

Regarding the stent biomaterials used, PLA was exclusively employed in five studies and combined with PCL in one study. Poly-L-lactic acid (PLLA) was used exclusively in three studies, combined with PDLLA in two studies, and combined with Mg alloy and PLGA in one study. Additionally, two studies exclusively used PLGA, three used PDX, one utilized poly(ε-caprolactone-co-lactide) (PCLA), and two employed Mg alloy alone. In five studies, a blend of multiple materials was used (Tables 1, 2, and 3).

Concerning degradation rates, only 11 articles provided specific information. The remaining articles focused on histological changes, stent placement techniques, and side effects.

The review identified three materials used in vascular stents: Mg alloy, a blend of PLA and PCL, and PLA alone. Magnesium alloy demonstrated a reported degradation rate of 28 days, while no degradation rates were provided for the other materials.

Ureteral stents were made from different materials and combinations. Magnesium combined with PLLA and PLGA showed the fastest degradation rate, breaking down in 35 days. PLLA combined with PDLLA had the slowest degradation rate, taking 120 days. For stents made from PGA combined with PLGA, the data only reported the start of degradation, which began after 28 days. Urethral stents were made from PDX and PLGA, with both materials exhibiting similar degradation rates of 84 and 90 days, respectively.

Biliary stents were made of Mg alloy, PLGA and PLLA. Magnesium alloy stents degraded within 60 days, PLGA had the fastest degradation at 35 days, and PLLA showed the slowest breakdown, taking up to 270 days.

Esophageal stents were made from PCLA and PDX. A study on PCLA reported a degradation rate of 28 days. For PDX, two retrospective studies did not provide exact degradation rates. The only study involving a cat, the PDX stent was completely gone during a follow-up 120 days after implantation. Another study reported that degradation of the PDX stent started around day 56.

Only one study involved the use of BDS in the trachea, and it was made of PLLA. However, the degradation rate was not reported. The mean degradation times of all materials are summarized in Table 4.

Complications were reported in 11 articles (52.4%), all of which involved minor and mostly self-limiting issues. In studies on biliary stents, two reported an initial increase in liver enzyme levels and two observed mild inflammation of the choledochal duct wall [19, 23]. In addition, one study noted anorexia and jaundice within the first 4 weeks [24]. For esophageal stenting, complications included stent migration [21], reduced cervical esophageal motility [8], and restenosis in three out of six cases [7]. The only study on tracheal stenting reported significant granulation tissue formation at the distal end of the stent in one dog, which was attributed to incomplete laceration of the membranous portion of the trachea [32]. In urinary stenting, complications included hydronephrosis in one case, associated with the implantation technique [16], death due to anesthetic complications in one study [30], mild epithelial hyperplasia in another [30], and hematuria and pain in five cases, both managed successfully with proper analgesia [22]. Vascular stenting studies reported no complications.

## 4. Discussion

Scoping reviews are particularly valuable when dealing with complex areas that lack comprehensive reviews [33]. This scoping review aimed to summarize studies on the use BDS in companion animals, identifying common themes, trends, and gaps in the literature. The findings highlighted limited evidence in the veterinary field, with 90% of research in this area conducted within human medicine, using animal models for translational purposes.

To ensure a rigorous and systematic review process, this scoping review followed the PRISMA-ScR guidelines and adhered to the Cochrane Collaboration's recommendations. The PRISMA-ScR framework enhanced transparency and comprehensiveness in reporting, enabling a thorough assessment of the search strategy and findings. Meanwhile, the Cochrane Handbook provided a robust methodological foundation, minimizing bias and ensuring the inclusion of all relevant studies. This approach resulted in reliable, reproducible, and trustworthy findings.

While these studies focus primarily on human medicine, they provide valuable insights that should not be overlooked. With most reporting a low incidence of minor, self-limiting complications, they offer an opportunity to explore and assess the application of BDSs in real-world cases involving companion animals.

In companion animals, non-BDS have been widely used to treat ureteral blockages. Although these stents have shown positive outcomes, their use is often associated with a high incidence of lower urinary tract signs, which can limit their clinical utility [6, 34]. In this scoping review, BDS were generally well tolerated. The risk of ureteral obstruction due to stent degradation is a major concern in human medicine [35]. Among the 113 dogs included, only one case of ureteral obstruction was reported following the placement of a BDS [16].

Urethral non-BDS has been used in companion animals, in conditions such as chronic mucosal edema, functional idiopathic urethral obstruction, or urethral wall disruption and in oncological cases where tumors either directly affect the urethra or compress it externally due to their size [6]. For clinical cases requiring short-term stenting, BDSs present a promising alternative. Unlike non-BDS, which are associated with complications such as mild to severe incontinence, stranguria, secondary urinary tract infections, stent migration, or fracture [6], BDSs have shown fewer adverse effects. In this review, 34 dogs treated with urethral BDS experienced only mild hematuria and some discomfort, both of which were effectively managed within a week [22]. These findings demonstrate the safety and feasibility of BDSs for patients with urethral or ureteral trauma or obstructions, effectively preventing cicatricial stenosis. BDS eliminates the need for a second surgery to remove the stent and reduces the risk of stent fracture.

Regarding the use of esophageal BDS, it was studied in a sample of 11 animals in which 4 were only used to evaluate the stent shape response to body temperature. In human applications context, the use of BDS in cases of esophageal stenosis is primarily intended to offer temporary relief, with the expectation that the stricture will resolve or that the stent will be replaced if the stricture persists or recurs after the stent dissolves. In the study conducted by Lam and colleagues [7] on esophageal stenosis in dogs, it was observed that half of the patients experienced restenosis within 2 or 3 months. The BDS used in the study were originally designed for human applications and are prone to losing their mechanical strength after about 3 weeks, with structural disintegration starting within 8 weeks. Given this constraint, it would be advisable to investigate this approach on a larger sample size and with a different type of biodegradable material in order to pursue improved outcomes.

Obstruction of the choledochal duct in companion animals can arise from intramural, extramural, or intraluminal causes and may lead to serious complications, including gallbladder rupture [33]. Several treatments have been proposed, including biliary decompression through percutaneous ultrasound-guided cholecystocentesis, open surgical procedures like cholecystoenterostomies, or the placement of a choledochal stent [34]. Biliary rerouting procedures, such as cholecystoenterostomies, are complex and carry certain risks. In contrast, choledochal stenting provides a less invasive and technically simpler alternative that preserves the biliary tract's anatomy and may reduce perioperative and postoperative complications. Given that many causes of obstructions are transient and do not require permanent stent placement, the use of BDS emerges as an attractive and viable option. Complications reported were limited to transient increases in liver enzymes and a single case of anorexia, which resolved with supportive care. Since biliary obstructions are common in both dogs and cats, and with several cases already reported where biliary stents have been used to treat this condition, the use of BDS could be an interesting area for further study [36–38]. As observed in humans, prolonged use of non-BDS can lead to stent obstruction and the need for removal [35].

Only one study reported on tracheal BDS [39]. In companion animals, the tracheal and bronchial non-BDSs are widely used in cases of tracheal and bronchial collapse [40]. In these cases, the idea is to use a permanent stent, so BDS would not be the best option.

The present review also illustrates safety in the use of vascular BDS, no complications or side effects were described in 61 patients. In humans, the primary indications for BDS are occlusion and stenosis of coronary arteries, peripheral arteries, and even veins [2]. However, in companion animals, these conditions are not frequently described, limiting the utilization of BDS in such cases.

Considering the biomaterial applied, nine polymers and copolymers were identified. In veterinary medicine, one of the main field biodegradable materials are used is to produce absorbable sutures. PGA, PLGA, PDX, and PCLA are easily found in commercial sutures brands; these materials are widely studied and well tolerate and can be found in different degradation rate according with their composition [41]. This scoping review did not find any great complication associated to any of these polymers: use of BDS made of PGA did not show any complications or side effects [14]. PLGA base biliary stents only showed a temporary elevation of liver enzymes after surgery [19], and PDX-based urethral stent reveals mild and temporary hematuria [22]. Regarding the urinary system, it remains inconclusive whether the described side effects were attributed to the physical presence of the stent or the specific material employed; either way, both had self-limiting symptoms that did not influence the outcome. PDX-based esophageal stent presented hyperplastic ingrowth tissue in one of six patients [7], and once more, we cannot determine if the reaction was because of the PDX material or the physical presence of the stent or the stent design. PCLA-based esophageal stent did not show any local reactions and in one of 4 dogs, the stent migrated [21]. Again, we are unable to ascertain whether the observed outcome is attributed to the design of the stent or the presence of PCLA within the stent. Mg alloy stents were also well tolerated with no major complications [23, 27].

This scoping review has some limitations. Despite using a variety of free text and MeSH terms to capture the use of BDS in companion animals, some studies may have been missed due to alternate terminology. In addition, database selection bias could have excluded certain studies. However, the risk of overlooking relevant research is minimal due to our thorough manual review, including the examination of reference lists in the included articles. Long-term outcome evaluation is limited, as most animals were euthanized at the conclusion of the studies. In addition, since 90% of the studies relied on animal models, the findings do not fully reflect outcomes in real clinical cases. This highlights the need for high-quality research to determine the optimal type and design of BDS for different pathologies and species based on real clinical cases.

Lange and collaborators affirm that the stent of the future will be degradable, in a control manner, and possible to coat or elute active compounds [37]. By shedding light on the existing knowledge and identifying any gaps, this research sets the stage for further advancements and informed decision-making in the use of BDS in companion animal's medicine.

## Figures and Tables

**Table 1:** Main results of the application of biodegradable stents in the urinary tract (urethra and ureter) of companion animals (9 studies; 147 animals).

Placement site	Biomaterial	Animals	Outcome	Reference
Ureter	PGA + PLGA	24 dogs	The stent provided temporary renal drainage as good as commercially available biostable stents with good biocompatibility and physical characteristics.	[14]
Ureter	PLLA + PDLLA	18 dogs	This biodegradable ureteral stent was more advantageous than the double-J stent for treating ureteral injury in a canine model.	[15]
Ureter	SR-PLA 96	12 dogs	SR-PLA 96 ureteral stent showed more favorable antireflux properties than a double-J stent.	[16]
Ureter	PLLA + PLGA + magnesium	18 dogs	The drainage of the novel stent is similar to the conventional; the biocompatibility and antibacterial ability of the novel stents are better than the conventional stents.	[17]
Ureter	SR-PLA 96	16 dogs	The risk of pressure-induced kidney damage was lowered, and the risk of upper urinary tract infections is reduced.	[18]
Ureter	SR-PLA 96	16 dogs	The stent is highly compatible, and SR-PLA 96 might be suitable material for a partial ureteric stent.	[19]
Ureter	PLLA + PDLLA	9 dogs	The renal concentration capacity was effectively protected and the half-time of kidney washout was shortened in the kidney that was stented.	[20]
Urethra	PGA + PLA	25 dogs	The biocompatibility of SR-PGA and SR-PLA stents was good when combined with Nd: YAG laser treatment of the prostate.	[21]
Urethra	PDX	9 dogs	Acceptable inflammatory reaction with gradually increasing granulation tissue but no luminal obstruction within 12 weeks.	[22]

*Note:* PDLLA: poly(D-lactic), PDX: poly(p-dioxanone), PLGA: poly(lactic-coglycolic acid), PLLA: poly(L-lactic).

Abbreviations: PGA, poly(glycolic acid); PLA, poly(lactic acid); SR-PLA, self-reinforced polylactic acid.

**Table 2:** Main results of the application of biodegradable stents in the gastrointestinal tract (choledochal duct and esophagus) of companion animals (7 studies; 32 animals).

Placement site	Biomaterial	Animals	Outcome	Reference
Choledochal duct	Magnesium	6 dogs	This stent has better mechanical properties than the nitinol stent and have good biocompatibility.	[23]
Choledochal duct	PLLA	3 dogs	Stent degradation was macroscopically evident in all animals at 9 months and was proven by SEM in 2 dogs at 6 months and in all of them at 9 months.	[18]
Choledochal duct	PLGA	6 dogs	The results showed that the PLGA stents exhibited the required biomedical properties and spontaneously disappeared from common choledochal duct s in 4–5 weeks	[19]
Choledochal duct	PLLA	3 dogs	The stent had good biocompatibility, self-clearing effect to clear the attached sludge away. The self-expanding property facilitated stent implantation and suggested possibility to be implanted endoscopically.	[24]
Esophagus	PCLA	4 dogs	The implanted deformed stent could be triggered by body temperature and expectedly returned to a nearly round shape to support esophageal wall.	[21]
Esophagus	PDX	9 dogs	Esophageal stenting is not a safe and efficacious procedure for treatment of recurrent benign esophageal stricture.	[7]
Esophagus	PDX	1 cat	Fluoroscopic examination revealed that the stent radio-opaque markers had gone, and the cervical esophagus was no longer air filled. The cervical esophagus was found to be freely distensible.	[8]

*Note:* PLLA: poly(L-lactic), PLGA: poly(lactic-coglycolic acid), PCLA: poly(ε-caprolactone-co-lactide), PDX: poly(p-dioxanone).

Abbreviation: SEM, scanning electron microscope.

**Table 3:** Main results of the application of vascular biodegradable stents in companion animals (4 studies; 61 individuals).

Placement site	Biomaterial	Animals	Outcome	Reference
Common iliac artery	PLA	3 dogs	Increasing neointimal formation and reduced patency during early follow-up	[25]
Coronary and femoral artery	Magnesium	40 dogs	Angiography of coronary artery and femoral artery confirmed that the lumen was clear and there were no elastic recoil and thrombosis. Stents completely disappeared 7 days after implantation.	[26]
Common iliac artery	PLA	1 dog	Preliminary measurements are necessary to estimate the reliability of quantitative histomorphometry. Measurements should be made and reported before final results are given.	[27]
Carotid artery	PLA + PCL	17 dogs	BDNCS may be a useful approach for aneurysm occlusion. However, mild in-stent stenosis was relatively high.	[28]

*Note:* PCL: poly(ε-caprolactone).

Abbreviations: BDNCS, biodegradable nanofiber-covered stent; PLA, poly(lactic acid).

**Table 4:** Average degradation time (days) reported in the different biodegradable stents according to the placement site and used biomaterial.

Placement site	Biomaterial	Mean degradation time (days)	Reference
Vessels	Magnesium alloy	28	[26]
	PLA + PCL	N/A	[28]
	PLA	N/A	[25, 27]

Ureters	PLLA + PLGA + magnesium	35	[17]
	PGA + PLGA	N/A	[14]
	PLA	84	[16, 29, 30]
	PLLA + PDLLA	120	[15, 20]

Urethra	PDX	84	[22]
	PLGA	90	[31]

Choledochal duct	Magnesium	60	[23]
	PLGA	35	[19]
	PLLA	180	[18, 24]

Esophagus	PCLA	28	[21]
	PDX	N/A	[7, 8]

Trachea	PLLA	N/A	[32]

*Note:* PCL: poly(ε-caprolactone), PCLA: poly(ε-caprolactone-co-lactide), PDLLA: poly(D-lactic), PDX: poly(p-dioxanone), PLGA: poly(lactic-coglycolic acid), PLLA: poly(L-lactic).

Abbreviations: PGA, poly(glycolic acid); PLA, poly(lactic acid).

## Data Availability

The data that support the findings of this study are available from the corresponding author upon reasonable request.
